# Etiology of acute gastroenteritis among children less than 5 years of age in Bucaramanga, Colombia: A case-control study

**DOI:** 10.1371/journal.pntd.0008375

**Published:** 2020-06-30

**Authors:** Ana E. Farfán-García, Aamer Imdad, Chengxian Zhang, Mónica Y. Arias-Guerrero, Nayibe T. Sánchez-Álvarez, Junaid Iqbal, Adriana E. Hernández-Gamboa, James C. Slaughter, Oscar G. Gómez-Duarte

**Affiliations:** 1 Facultad de Ciencias de la Salud, Grupo de Investigación en Manejo Clínico –CliniUDES, Programa de Bacteriología y Laboratorio Clínico, Universidad de Santander, Bucaramanga, Colombia; 2 Division of Pediatric Gastroenterology, Department of Pediatrics, SUNY Upstate Medical University, Syracuse, New York, United States of America; 3 Division of Pediatric Infectious Diseases, Department of Pediatrics, Vanderbilt University School of Medicine, Nashville, Tennessee, United States of America; 4 Facultad de Ciencias de la Salud, Grupo de Investigación en Manejo Clínico –CliniUDES, Maestría en Investigación en Enfermedades Infecciosas, Universidad de Santander, Bucaramanga, Colombia; 5 Fundación Oftalmológica de Santander-FOSCAL, Floridablanca, Colombia; 6 Department of Biostatistics, Vanderbilt University School of Medicine, Nashville, Tennessee, United States of America; 7 International Enteric Vaccine Research Program, Division of Pediatric Infectious Diseases, Department of Pediatrics, University at Buffalo; The State University of New York, Buffalo, New York, United States of America; Mohammed Bin Rashid University of Medicine and Health Sciences, UNITED ARAB EMIRATES

## Abstract

**Background:**

Acute gastroenteritis (AGE) is a major cause of morbidity and mortality in children aged less than 5 years in low- and middle-income countries where limited access to potable water, poor sanitation, deficient hygiene, and food product contamination are prevalent. Research on the changing etiology of AGE and associated risk factors in Latin America, including Colombia, is essential to understand the epidemiology of these infections. The primary objectives of this study were to describe etiology of moderate to severe AGE in children less than 5 years of age from Bucaramanga, Colombia, a middle-income country in Latin American, and to identify the presence of emerging *E*. *coli* pathotypes.

**Methodology/Principal findings:**

This was a prospective, matched for age, case-control study to assess the etiology of moderate to severe AGE in children less than 5 years of age in Bucaramanga, Colombia, South America. We tested for 24 pathogens using locally available diagnostic testing, including stool culture, polymerase chain reaction, microscopy and enzyme-linked immunoassay. Adjusted attributable fractions were calculated to assess the association between AGE and each pathogen in this study population. The study included 861 participants, 431 cases and 430 controls. Enteric pathogens were detected in 71% of cases and in 54% of controls (p = <0.001). Co-infection was identified in 28% of cases and in 14% of controls (p = <0.001). The adjusted attributable fraction showed that Norovirus GII explained 14% (95% CI: 10–18%) of AGE, followed by rotavirus 9.3% (6.4–12%), adenovirus 3% (1–4%), astrovirus 2.9% (0.6–5%), enterotoxigenic *Escherichia coli* (ETEC) 2.4% (0.4–4%), *Cryptosporidium sp*. 2% (0.5–4%), *Campylobacter sp*. 2% (0.2–4%), and *Salmonella sp*.1.9% (0.3 to 3.5%). Except for *Cryptosporidium*, all parasite infections were not associated with AGE. Three emergent diarrheagenic *E*. *coli* pathotypes were identified in cases (0.7%), including an enteroaggregative/enterotoxigenic *E*.*coli* (EAEC/ETEC), an enteroaggregative/enteropathogenic *E*.*coli* (EAEC/EPEC), and an emergent enteroinvasive *E*. *coli* with a rare O96:H19. No deaths were reported among cases or controls.

**Conclusions/Significance:**

Norovirus and rotavirus explained the major proportion of moderate to severe AGE in this study. Higher proportion of infection in cases, in the form of single infections or co-infections, showed association with AGE. Three novel *E*. *coli* pathotypes were identified among cases in this geographic region.

## Author summary

Acute gastroenteritis (AGE) is a leading cause of mortality in children under 5 years of age in low- and middle-income countries (LMIC). The highest burden of AGE disease is concentrated in tropical areas where populations lack access to clean water, adequate sanitation and hygiene, making this condition a neglected disease. Limited information on etiology, associated malnutrition, and mortality among underserved communities makes difficult the development of strategies for AGE prevention and treatment. This case-control study among children less than 5 years of age in Bucaramanga, Colombia, revealed that viral followed by bacterial organisms explained the larger proportion of AGE, being norovirus the most common organism. The higher rate of infections and co-infections among cases compared to controls was associated with AGE. This study also reports the identification of three new *E*. *coli* pathotypes among cases designated as biofilm-forming enteroinvasive *E*. *coli* (BF-EIEC), enteroaggregative/enteropathogenic *E*. *coli*, and enteroaggregative/enterotoxigenic *E*. *coli* (EAEC/ETEC).

## Introduction

Acute gastrointestinal infections are a major source of morbidity and mortality in preschool children and lead to about 446,000 deaths every year, mainly in low and middle-income countries [1]. The highest burden is concentrated in tropical areas where populations lack access to clean water, sanitation and hygiene, making this condition a neglected disease [2]. The etiology of moderate to severe acute gastroenteritis (AGE) varies in different regions of the world and depends on multiple host and environmental factors [3–6]. Case-control studies have reported comprehensive data on the etiology of moderate to severe diarrhea from low-income countries in Africa and South Asia [3,5]. Colombia, a middle-income country in Latin America, has an estimated risk of AGE of 2.5% in children less than 5 years of age and risk of 2.0% in less than 10 years of age [7]. Studies reported that rotavirus remains a leading cause of AGE in Colombia together with norovirus, with a reduced hospitalization rate, thanks to the introduction of monovalent rotavirus vaccine in Colombia in 2009 [8–10]. Studies on *E*. *coli* diarrheagenic revealed that these agents are associated with AGE and they are common contaminants of food products for human consumption [11–17]. Information on the etiology of AGE in Colombia is based on surveillance studies with a limited number of pathogens tested and limited information on novel or emergent *E*. *coli* pathotypes, defined as *E*. *coli* containing virulence factors from more than one *E*. *coli* pathotype or classic *E*. *coli* pathotypes with new phenotypes. One example of these emergent pathogens includes the EAEC/STEC serotype O104:H4 German strain implicated in a severe outbreak of bloody diarrhea, and hemolytic uremic syndrome in 2011 in Germany and other European countries [18,19]. To identify risk factors among cases of AGE compared to controls and to evaluate the association of enteric pathogens with AGE, we conducted a case-control study in children less than 5 years of age in Bucaramanga, Colombia. The primary objectives of this study were to define the etiology of moderate to severe AGE in children less than 5 years old in Bucaramanga, Colombia, Latin America and to identify novel and emergent *E*. *coli* pathotypes.

## Materials and methods

### Study design and participants

This was a prospective, multi-center, age-matched, case-control study to determine the etiology of moderate to severe acute gastroenteritis (AGE) in children < 5 years of age. The study was conducted from July 2013 to December 2014. The details of methods for this study were published previously [20] and are presented in detail as supplementary data (Supplementary materials and methods). The study was conducted in Bucaramanga, Colombia, a city of about 525,000 inhabitants located on a plateau of the Andes at 959 meters above sea level. The city has an average temperature of 22°C and, based on the Koppen climate classification, it features a tropical monsoon climate [21]. The rotavirus vaccine is offered to all children in this city and the coverage is 79%, being 90% the average for Colombia [22,23]. The cases were recruited from five major urban medical centers from emergency, inpatient, and outpatient facilities. Controls were recruited from outpatient clinics only. Inclusion and exclusion criteria for cases and controls were described in Table 1. A case of AGE was defined as ≥ 3 loose stools or ≥ 1 vomiting episode in last 24 hours and duration of no more than 10 days before the day of enrollment. The definition of ‘moderate to severe’ case was AGE plus one of the following: sunken eyes, confirmed by parent/caretaker and researchers; loss of skin turgor by skin pinch (>1 second recovery); intravenous rehydration prescribed or administered; dysentery (1 or more bloody stools); evaluated in an emergency department (ED) or admitted to the hospital for AGE (See Table 1) [20]. A follow-up phone call was made at 2 weeks and 2 months to inquire about any health complication including death related to AGE-associated illness.

**Table 1:** Inclusion/Exclusion criteria for cases and controls.


Cases
Inclusion criteria
**Children less than 5 years**
**Presence of acute, moderate-to-severe diarrhea and/or vomiting within the past 10 days. Acute gastroenteritis was defined as 3 or more episodes of loose or liquid stools within 24 hours and or more than one episode of vomiting in 24h.**
**Children who are not part of this study as a case (not previously recorded to have diarrhea within the last 60 days)**
**AGE is moderate to severe, and must meet at least one of the following criteria.**
**Sunken eyes, confirmed by parent/caretaker**
**Loss of skin turgor by skin pinch (≤ 2s slow, or > 2 very slow)**
**Intravenous rehydration prescribed or administered**
**Dysentery (1 or more bloody stools)**
**Evaluated in the Emergency Department or admitted to the hospital for diarrhea**
**Children will still be eligible for enrollment even if they have received antibiotics within the last 14 days.**
**Exclusion criteria**
**Children older than 60 months of age**
**Children who reside outside of the metropolitan area of Bucaramanga, Colombia**
**Presence of chronic diarrhea (> 10 days) or other co-morbid conditions such as Crohn’s disease or ulcerative colitis.**
Controls
Inclusion criteria:
**Children less than 5 years**
**Child who resides within the metropolitan area of Bucaramanga, Colombia**
**Absence of diarrhea or vomiting within the past 10 days.**
**Matched to cases for age. Age matching is ±2 months for 0–11 months, ±4 months for 12–59 months (can not exceed the stratum boundaries of the case).**
**Exclusion criteria:**
**Children older than 60 months of age**
**Child who does not reside within the Metropolitan area of Bucaramanga, Colombia**
**Presence of acute diarrhea, as defined by the WHO, in the previous 7 days (regardless of whether they develop diarrhea after enrollment)**
**Presence of chronic diarrhea (> 10 days) or other co-morbid conditions such as Crohn’s disease or ulcerative colitis.**

### Data collection and procedures

Informed consent for subject participation was obtained from parents or guardians as all participants were children less than 5 years of age. Table 1 gives inclusion/exclusion criteria for cases and controls. Patients were recruited from emergency, inpatient and outpatient facilities of the above-mentioned medical centers. Controls were healthy children with no AGE for the last 10 days. They were recruited to match for age and treatment facility. After written informed consent was obtained, an interview questionnaire was administered to the subject’s parents or guardians at enrollment, and at 2 and 6 weeks after that. The interview was conducted and recorded in Spanish, by trained study clinical researchers. Information initially captured by interviewers in paper-based questionnaires was digitized and stored in the REDCap (Research Electronic Data Capture; www.project-redcap.org) database. REDCap is a secure, web-based application designed to capture data for research studies, providing an intuitive interface for validated data entry, audit trails for tracking data manipulation and export procedures for seamless data downloads to common statistical packages, and procedures for importing data from external sources [24].

At the time of enrollment, data was collected for demographics, medical history, nutritional history, water sources, and epidemiological and socioeconomic factors. Information about anthropometry was obtained from medical records. Stool samples were collected on the day of enrollment up to 1 week after enrollment. Stool samples, collected spontaneously from subjects in sterile disposable plastic bottles, were kept on ice and transported within 4 hours to the laboratory for processing the same day. Specific details on procedures are provided as supplementary data (Supplementary materials and methods).

### Microbiologic methods

A detailed description of the microbiologic and molecular methods were published previously as part of our AGE pilot study [20]. Standard stool culture techniques were applied to detect bacteriological pathogens including *Salmonella* spp., *Shigella* spp., *Campylobacter* spp., *Yersinia* spp. and *E*. *coli*. To identify *E*. *coli* pathotypes, DNA template of five individual *E*. *coli* isolates colonies per subject were tested for target genes with multiplex polymerase-chain reaction (mPCR) (S1 Fig, S1 and S2 Tables). Individual characterization of *E*. *coli* isolates was essential for detection of emergent *E*. *coli* pathotypes, defined as *E*. *coli* pathotypes containing virulence genes from more than one classic pathotype, and for detection of co-infections with individual pathotypes within the same subject’s stool specimen. The mPCR was designed to detect genes found in six well-established pathogenic *E*. *coli* pathotypes as follows: gene *daaE* for diffusely adherent *E*. *coli* (DAEC); *aaiC* and *aggR* for enteroaggregative *E*. *coli* (EAEC); *ipaH* for enteroinvasive *E*. *coli* (EIEC); *eae* and/or *bfpA* for enteropathogenic *E*. *coli* (EPEC); *lt* and/or *st* for enterotoxigenic *E*. *coli* (ETEC); and *stx* and/or *eae* for Shiga-toxin producing *E*. *coli* (STEC), as described before [20]. We further categorized EPEC as typical EPEC (*eaeA*+ *bfpA*+) or atypical EPEC (*eaeA*+ *bfpA*-) [20,25]. Also, EAEC were further subtyped in typical EAEC (*aggR+*) or atypical EAEC (*aggR-*, *aaiC+*) [26].

Reverse transcriptase PCR (RvT-PCR) was used to detect norovirus, astrovirus, and sapovirus, and adenovirus detection from DNA isolated from stool samples was performed by real-time PCR (RT-PCR) (S1 and S2 Tables) [13,27,28]. Enzyme-linked immune-assays (AccuDiag ELISA, Diagnostic Automation, Inc., Woodland Hills, CA) were used to detect rotavirus, *Giardia duodenalis*, *Entamoeba histolytica/E*. *dispar and Cryptosporidium* [20]. Stool samples were also evaluated by direct examination under bright-field microscopy for detection of alternative parasites including, *Endolimax nana*, *Chilomastix mesnili*, *Blastocystis hominis*, among others. Unused stool specimens were stored at -80°C for future studies. Similarly, bacterial clinical isolates were stored in Luria broth supplemented with 10% glycerol for future studies. Medical technology-certified professionals conducted microscopy examinations and parasite detection from stool specimens.

### Statistical analysis

Sample size calculations were initially based on a balanced design with 300 cases and 300 controls, which was subsequently enriched to 430 in each group to increase the number of potential emergent *E*. *coli* pathogens. The power to detect significant associations is impacted by the underlying prevalence of a pathogen. With 430 subjects in a group, we had 80% power to detect an odds ratio of 4.8 if the control prevalence was 1% and 80% power to detect an odds ratio of 1.7 if the control prevalence was 20%. All calculations used a 0.05 level of significance. All statistical tests were two-sided.

Continuously-measured baseline variables were described using mean (+/- SD) and compared by case/control status using the 2-sample t-test. The Mann-Whitney test was used when data were not normally distributed. Categorical variables were summarized using percentages and compared by case/control status using Pearson’s Chi-squared test. Unadjusted and adjusted multivariable logistic regression models were used to estimate the association of each pathogen with case status. Separate models were fit for each pathogen of interest. We adjusted for the same set of candidate confounders in each multivariable model. To mitigate bias due to overfitting, we decided *a priori* to include age, sex, nutritional status, and co-infection [20]. We further included health insurance, education of primary caregiver and handwashing, based on statistical significance after the univariate analysis. The results of logistic regression analysis were presented as odds ratios with 95% confidence intervals. Our prespecified analysis plan included subgroup analyses based on age groups and coinfection. The subgroup analysis for coinfection was considered for a pathogen when we observed at least 5 subjects with single infection and at least 5 subjects with coinfection. Because previous studies have shown that etiology and risk of mortality changes with age [3], we stratified subjects into the following age groups: <12 months, 12 to <24 months, and 24 to 59 months. Missing data was noticed and reported accordingly. *A priori*, we decided to do multiple imputations only if overall data were missing for at least 5% of study population. Since data were missing for less than 5% of the variables in the analysis, we did not perform multiple imputations.

We calculated the attributable fraction (risk) for each pathogen. Attributable risk is defined as the fraction of the total population that would not have occurred if the effect associated with the risk factor of interest was absent. Typically prospective studies are used to calculate risk and attributable risk; however, case-control studies can be used to calculate the same as described previously [29]. The assumptions considered were that the cases in the regression model can be considered a random sample of all cases in the population and that the disease is “rare” (i.e. it has an incidence of < 10%). All statistical analyses were performed using STATA 14 [30].

### Ethics statement

The institutional review boards (IRB) at the at the University at Buffalo (IRB STUDY000007960), Vanderbilt University (IRB 130327), Universidad de Santander and each participating medical center in the metropolitan area of Bucaramanga, approved the study. A written informed consent in Spanish was obtained from parents or guardians of all participants.

## Results

### Demographics of study population

**Fig 1:**
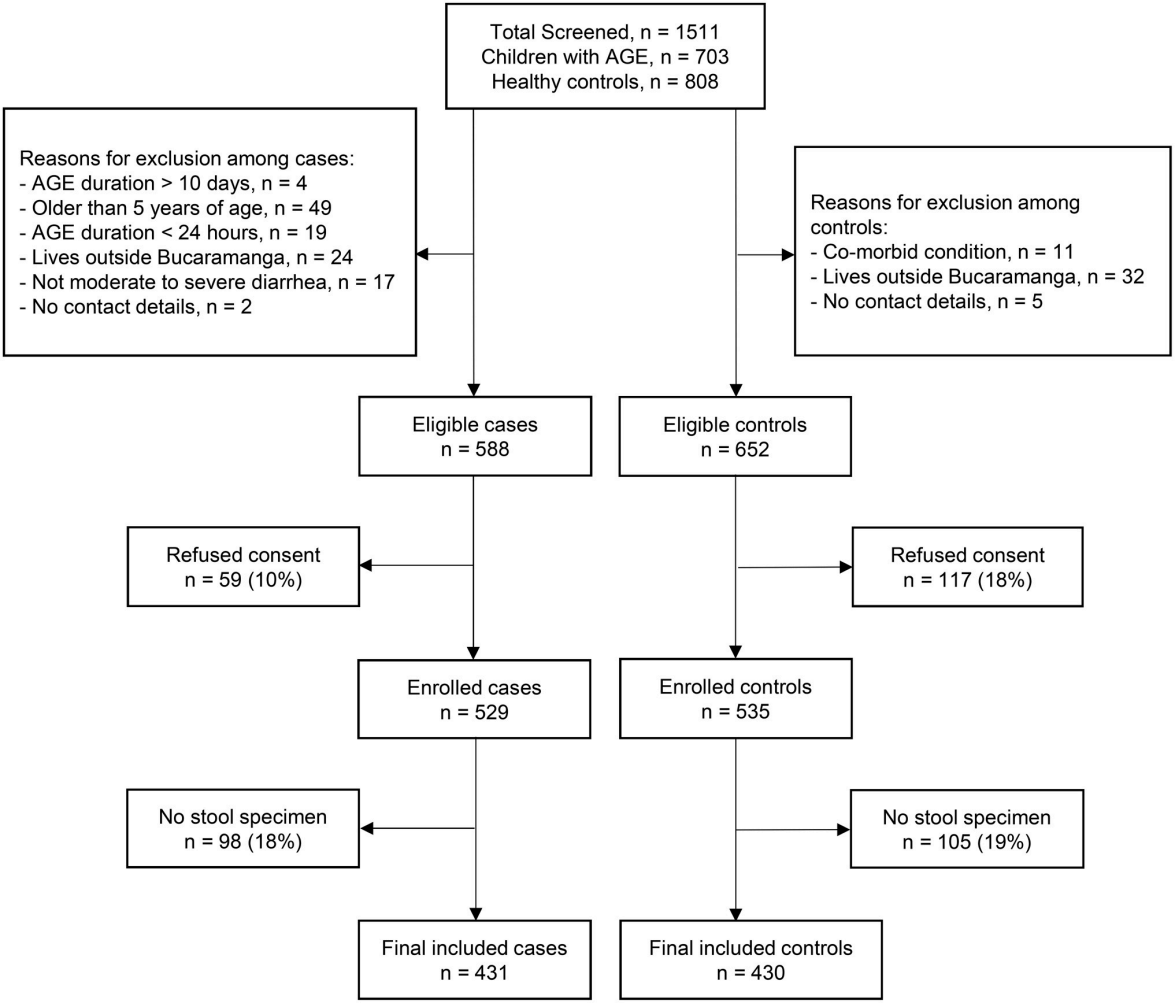
Recruitment of cases of moderate to severe AGE and healthy controls in the metropolitan area of Bucaramanga, Colombia. Cases were children with AGE and controls were healthy children. Abbreviation: AGE, acute gastroenteritis.

During the 18 months study period, 1,511 children were screened and 861 (57%) agreed to participate, including 431 cases of AGE and 430 controls (Fig 1). Sixteen percent of children with AGE and 19% of healthy controls had parents or guardians that refused to participate (Fig 1). The baseline characteristics were similar in terms of age, gender, race, income, caregiver education and nutritional status (Table 2). In contrast, handwashing with water and soap was more common in controls (99%) than cases (95%) with a *p*-value = .007, and exposure to sick contacts with diarrhea was more common in cases (17%) than controls (7%) with a *p*-value = < .00 (Table 2). We recruited 12% (52/431) of cases from inpatient settings and the remaining were recruited from either outpatient clinics or emergency departments.

**Table 2:** Demographic and epidemiological factors among cases and controls.

Variable	Cases (431) n (%)	Controls (430) n (%)	*p*- value
**Age: 0–<12 months**	**140 (32)**	**139 (32)**	**0.945**^g^
**Age: 12–<24 months**	**148 (35)**	**152 (36)**	
**Age: 24–59 months**	**143 (33)**	**139 (32)**	
**Male**	**235(54)**	**225(52)**	**0.517**
**Race, White**	**190(44)**	**171(40)**	**0.199**
**Race, Mestiza**^a^	**241(56)**	**259(60)**	
**History of preterm birth**	**73(16)**	**54(12)**	**0.070**
**Breastfeeding**	**414(96)**	**414(96)**	**0.864**
**Health Insurance**	**425(98)**	**424(98)**	**0.996**
**Household Income per month in Cop**^b^			**0.457**
** <589, 500 (<US$200)**	**123(30)**	**128(31)**	
** 589,500 to 1,179,000 (US$200–393)**	**252(61)**	**250(62)**	
** 1,180,000 to 3,534,000 (US$394–1,178)**	**38(9)**	**28(7)**	
**Family own a house**	**281(65)**	**263(61)**	**0.219**
**Education of Primary caregiver**^c^			
**Completed elementary school**	**349 (81)**	**368 (85)**	**0.701**
**Access to clean water**	**276(64)**	**279(64)**	**0.795**
**Refrigerator available**	**418(96)**	**417(96)**	**0.995**
**Handwashing with soap and water**	**410(95)**	**423(99)**	**0.007**
**Sick contacts at home**	**75(17)**	**31(7)**	**0.000**
**Rotavirus vaccine**	**409(96)**	**404(94)**	**0.546**
**Underweight**^d^^,^^e^	**16 (3)**	**16 (3)**	**0.994**
**Stunting**^f^	**47 (11)**	**49 (11)**	**0.819**
**Antibiotics prior to enrollment**	**50 (12)**	ND^h^	**-**

The percentages are in reference to total at the top of the column. P values obtained by Chi 2 test

Abbreviations: SD = standard deviation; kg = kilogram cm = centimeter; Cop: Colombian peso.

^a^ Mestiza is the ethnic category people with white and indigenous ancestry.

^b^Data missing for 7 (0.8%) participants;

^c^ One US dollar is equivalent to $3,000 Colombian pesos (Cop);

^d^Underweight: weight for age < –2 standard deviations (SD) of the WHO Child Growth Standards median. Data missing for 4 (0.4%) participants;

^e^ Data missing for 13 (1.5%) of participants

^f^ Stunting: height for age < –2 SD of the WHO Child Growth Standards median. Data missing for 32 (3.7%) of participants.

^g^The average age for cases was 655 days (449 standard deviation (SD)) and for controls was 611 (454 SD) with a *p*-value of 0.160.

^h^No data on antibiotics use prior to enrollment in healthy controls.

### Clinical features of AGE cases

A total of 431 children with moderate to severe AGE were enrolled in this study in Bucaramanga, Colombia. Diarrhea [(93% (n = 403)] and vomiting [71% (n = 305)] were the most common clinical manifestations among cases as these were part of the inclusion criteria. The proportion of diarrhea, vomiting, and fever was similar in all age groups (Table 3). The rate of abdominal pain reporting was higher in older age groups compared with younger age group (Table 3). Duration of diarrhea was less than 14 days among 410 (95.1%) of cases children, only 21 cases had diarrhea that went beyond the 2 week period. A total of 156 cases (36%) required intravenous fluid hydration as part of AGE treatment. A total of 55 cases (13%) required hospitalization for management of AGE. The 55 subjects that were hospitalized included 20 (6.4%) children <12 months of age; 16 (29.1%) from 12 to <24 months, and 19 (34.5%) from 24 to <60 months. Reasons for hospitalization included predominantly severe dehydration requiring IV fluids and infection requiring antibiotics. From a total of 55 hospitalized cases, 44 (80%) cases had severe dehydration, and 31 (56%) of them had evidence of systemic infection and dehydration. Dehydration was recorded among 393 (91%) of cases based on clinical manifestations (sunken eyes, decreased skin turgor) during enrollment. No deaths were reported among cases or controls at 14 days and 60 days follow up from day of enrollment.

**Table 3:** Clinical manifestations among cases of AGE at time of enrollment.

Clinical manifestations	0–<12 months (140)^a^		12–<24 months (148)		24–<60 months (143)		*p*-value^d^
	n	%	n	%	n	%	
Diarrhea^b^	137	31.8	141	32.7	125	29.0	<0.00*
Vomiting^b^	77	17.9	108	25.1	120	27.8	<0.00*
Fever	105	24.4	112	26.0	110	25.5	0.92
Abdominal pain	39	9.0	63	14.6	116	26.9	<0.00*
Dysentery^c^	42	9.7	27	6.3	27	6.3	0.03

^a^Total number of subjects in this age category.

^b^These variables are part of the inclusion criteria for cases.

^c^Dysentery was defined as presence of blood with or without mucus in the stool per parent or guardian description.

^d^Categorical *p*-value calculated by chi-square test.

*Values are statistically significant

### Infections and co-infections among cases and controls

Subjects in the study, including cases or controls, had stools evaluated for enteric pathogens. Enteric pathogen detection on stool samples revealed that 539 (63%) subjects were positive for at least one enteric pathogen among a total of 861 subjects, among them, 307 (71%) were cases and 232 (54%) were controls (*p*-value <0.001) (S3 Table). Co-infections were detected in 183 (21.3%) subjects, 123 (28.5%) among cases and 61 (14.2%) among controls (*p*-value: <0.001) (S3 Table). The number of pathogens detected per stool specimen among subjects varied from zero to a maximum of 4 (Table 4). The absence of pathogens among 28.8% of cases and 46% of controls showed association of no pathogen detection with control subjects. A total of 184 (43%) cases and 172 (40%) controls were infected by a single pathogen. The presence of two or more pathogens was predominantly associated with cases (Table 4).

**Table 4:** Proportion of cases of AGE and healthy controls with increasing number of pathogens identified in stools.

Pathogens	Cases (n- 431)		Controls (n-430)		*p-*value
	n	%	n	%	
None	124	28.8	198	46	<0.001
One	184	42.7	172	40	
Two	100	23.2	51	11.9	
Three	18	4.2	9	2.1	
Four	5	1.2	0	0.0	

Abbreviations. n: number; %: percentage; NA: not applicable.

The percentages are in reference to total at the top of the column. *p-*values obtained by Chi 2 test.

As chi square cannot be calculated when one of the cells values is zero, we assume a single control with 4 pathogens.

To evaluate the association of co-infection and AGE, we evaluated the possible association of different organisms with AGE cases when detected as a single infection or as a co-infection. Most of the pathogens detected in cases versus controls had a similar odds ratio as a single infection or as co-infection (S4 Table). In contrast, *Cryptosporidium*, astrovirus, and EAEC had a stronger association with cases when present in combination with other pathogens.

**Fig 2:**
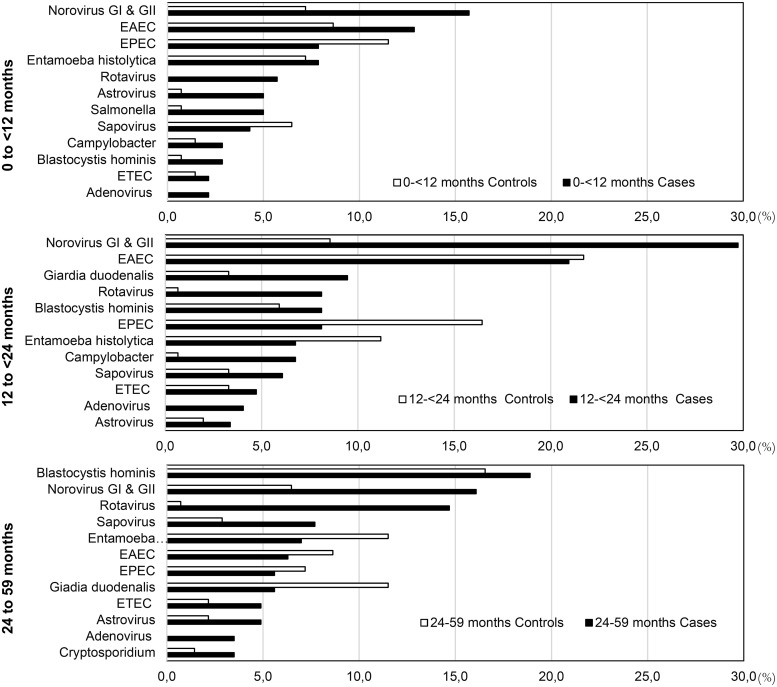
Proportion of diarrheagenic pathogens among cases of moderate to severe AGE and healthy controls stratified by age. Age stratification in months includes: 0 to <12, 12 to <24, and 24 to 59 months of age. Abbreviations: AGE, acute gastroenteritis; EAEC, enteroaggregative *E*. *coli*; EPEC, enteropathogenic *E*. *coli*; ETEC, enterotoxigenic *E*. *coli*.

The most common enteric pathogens among cases were norovirus with 89 (21%), EAEC with 60 (14%) and *Blastocystis hominis* with 43 (10%) (Table 5). The most common enteric pathogens among controls were EAEC with 57 (12%), EPEC with 51 (12%) and *E*. *histolytica* with 43 (10%) (Table 5). The most frequent pathogens detected as single infections among cases were norovirus (8%), EAEC (5%), and rotavirus (4%), and among controls were EAEC (9%), EPEC (7%), and norovirus (5%) (S4 Table). To evaluate the role of subject age on enteric pathogens detection among cases and controls, we stratified subjects by age and calculated the number of the most common enteric pathogens. Accordingly, the most common pathogens detected were as follows: i) in cases <12 months of age, norovirus, EAEC and EPEC; ii) in cases 12 to <24 months of age, norovirus, EAEC and *G*. *duodenalis*; and iii) in cases 24 to 59 months of age, *Blastocystis*, norovirus, and rotavirus were the most common (Fig 2).

**Table 5:** Prevalence of microorganisms in cases and controls and their association with moderate to severe diarrhea.

Pathogen	Case n = 431	Control n = 430	Total N = 861	Crude Odds Ratios	Adjusted Odd Ratios
**Norovirus (all subtypes), n (%)**	**89(21)**	**32(7)**	**121(14)**	**3.2 (2.10–4.9)**	(2.3–5.8)*
• **Norovirus GI, n (%)**	**15(3)**	**15(3)**	**30 (3)**	**0.99 (0.48–2)**	**0.98 (0.45–2)**
• **Norovirus GII, n (%)**	**73(17)**	**17(4)**	**90 (10)**	**4.9 (2.8–8.5))**	5.8 (3.2–10.2)*
• **Norovirus GI/GII, n %**	**1(0.2)**	**0 (0)**	**1 (0.1)**	**-**	**-**
**EAEC, n (%)**	**60(14)**	**57(12)**	**117 (14)**	**1.05 (0.72–1.56)**	**1.04 (0.67–1.62)**
**EPEC, n (%)**	**31 (7.2)**	**51(12)**	**82 (10)**	**0.59(0.37–0.94)**	**0.55 (0.33–0.91)**
***Blastocystis hominis***,^#^ **n (%)**	**43(10)**	**33(8)**	**76(8)**	**1.3 (0.82–2.14)**	**1.4(0.85–2.3)**
***Entamoeba histolytica*, n (%)**	**31(7)**	**43(10)**	**74(8)**	**0.68 (0.43–1.13)**	**0.68(0.39–1.17)**
***Giardia duodenalis* n (%)**	**24(6)**	**25(6)**	**49 (5.6)**	**0.95 (0.53–1.7)**	**0.91(0.49–1.7)**
**Sapovirus, n (%)**	**26(6)**	**18(4)**	**44(5)**	**1.4 (0.79–2.7)**	**1.3(0.71–2.6)**
**Rotavirus, n (%)**	**41(9)**	**2(0.4)**	**43(5)**	**22.5 (5.4–93.6)**	25(6.1–107)*
**ETEC, n (%)**	**17(4)**	**10(2)**	**27(3)**	**1.72(0.78–3.81)**	**1.96 (0.89–4.31)**
**Astrovirus, n (%)**	**19(4)**	**7(2)**	**26(3)**	**2.7 (1.15–6.6)**	2.9(1.1–7)*
***Campylobacter* spp, n (%)**	**15(3)**	**5(1)**	**20(2)**	**3 (1.10–8.5)**	3.2(1.0–9)*
**Adenovirus, n (%)**	**14(3)**	**1(0.2)**	**15(2.0)**	**14.4 (1.8–110)**	19.1(2.5–145)*
***Cryptosporidium* spp, n (%)**	**12(3)**	**3(0.7)**	**15(1.7)**	**4.0 (1.15–14.5)**	4.9 (1.3–17)*
***Salmonella* spp, n (%)**	**12(3)**	**2(0.4)**	**14(1.6)**	**6.12 (1.3–27.5)**	5.8 (1.2–27)*
**DAEC, n (%)**	**4(0.9)**	**2(0.4)**	**6(0.7)**	**2.0(0.36–11.0)**	**2.7 (0.44–16)**
**EIEC, n (%)**	**1(0.2)**	**2(0.2)**	**3(0.4)**	**0.49(0.04–5.50)**	**1.12 (0.07–16)**
***Endolimax nana***,^#^ **n (%)**	**3(0.7)**	**3(0.7)**	**6(0.7)**	**0.99 (0.2–4.9)**	**0.95 (0.1–7.9)**
***Entamoeba hartmanni***,^#^ **n (%)**	**3(0.7)**	**1(0.23)**	**4 (0.4)**	**3.0 (0.31–29.02)**	**2.6 (0.24–28)**
***Chilomastix mesnili***, ^#^ **n (%)**	**1(0.2)**	**3(0.7)**	**4(0.4)**	**0.33 (0.03–3.19)**	**0.36(0.04–3)**
Emergent ***E***. ***coli* Pathotypes, n (%)**	**4(0.9)**	**0(0)**	**4(0.5)**	**-**	**-**
***Yersinia enterocolitica*, n (%)**	**3(0.7)**	**0.0(0)**	**3(0.3)**	**-**	**-**
**STEC, n (%)**	**3(0.7)**	**0(0)**	**3(0.3)**	**-**	**-**
***Shigella* spp, n (%)**	**1(0.2)**	**1(0.2)**	**2(0.2)**	**0.99 (0.06–16.0)**	**1.2(0.05–29)**
***Entamoeba coli***, ^#^ **n (%)**	**1(0.2)**	**0.0(0)**	**1 (0.1)**	**-**	**-**

The percentages are will reference to totals given at the top of each column. Odd ratios obtained by logistic regression and results in the last column of the table are adjusted for age, sex, weight, insurance, education of primary caregiver, handwashing and presence of other pathogen. The results are presented for only those pathogens that had at least one positive test on cases and controls.

The total percentage in columns may exceed 100 as multiple pathogens were present in a single subject.

**Abbreviations:** DAEC, diffusely adherent *E*. *coli*; EAEC, enteroaggregative *E*. *coli*; EIEC, enteroinvasive *E*. *coli*; EPEC, enteropathogenic *E*. *coli*; ETEC, enterotoxigenic *E*. *coli*; and STEC, Shiga toxin producing *E*. *coli*.

*: OR and CI values above 1.0 that indicate association with AGE.

^#^ Indicates organisms not recognized as pathogens.

### Enteric organisms associated with AGE

Norovirus was detected in 21% of cases and 7% of controls and was significantly associated with moderate to severe AGE (OR 3.6, 95% CI 2.3–5.8) (Table 5). Other organisms significantly associated with moderate to severe AGE included rotavirus, astrovirus, adenovirus, *Campylobacter* spp., *Salmonella* spp., ETEC and *Cryptosporidium* (Table 5).

**Fig 3:**
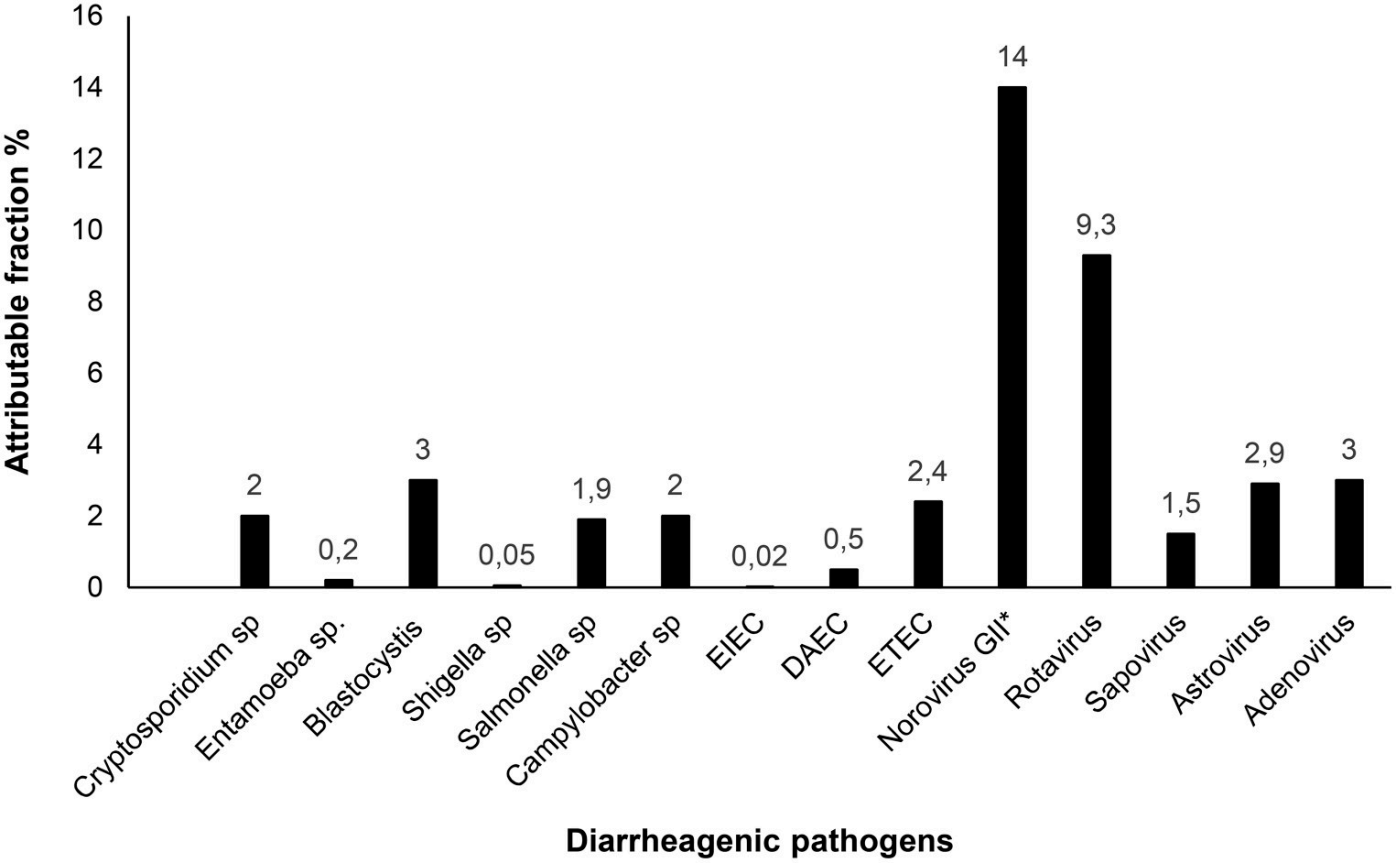
Attributable fraction for moderate to severe AGE for different diarrheagenic pathogens. Attributable fractions for different pathogens for moderate to severe gastroenteritis were calculated based on odd ratios calculated from adjusted models. Abbreviations: AGE, acute gastroenteritis; EIEC, enteroinvasive *E*. *coli*; DAEC, diffusely adherent *E*. *coli*; ETEC, enterotoxigenic *E*. *coli*.

The attributable fraction was calculated for selected pathogens that had a positive association (OR >1) with AGE. Norovirus had the highest attributable fraction (14%, 95% CI 10–18%) followed by rotavirus (9.3%, 95% CI 6–12%). ETEC, *Campylobacter* and *Salmonella* had the highest attributable fractions among bacteria (Fig 3). When data were analyzed separately for norovirus genotype I and II, only genotype II was significantly associated with moderate to severe AGE, even though numbers were smaller for norovirus GI [n = 30, (3%)] compared to norovirus GII [n = 90 (10%)] (Table 5). A subgroup analysis based on age, and coinfection for all pathogens, revealed similar results. Intestinal parasites were detected in 28% of cases and 26% of controls. Except for *Cryptosporidium*, the remaining enteric parasites detected in this study were not associated with AGE. Overall, 29% of AGE cases had undetected enteric pathogens. In 35% of cases, enteric pathogens detected were not associated with AGE at the single pathogen level when compared to controls. In the remaining AGE cases, viral infections explained 30% and bacterial infections explained 6% of the burden of moderate to severe AGE in this study population.

### Diarrheagenic *E*. *coli* pathotypes were common among cases and controls

*E*. *coli* pathotypes were the most common diarrheagenic agents with detection rates of 28% among cases and 26% among controls (Table 5, S6 Table). Similar proportion of typical EAEC (those containing *aggR*) and atypical EAEC (those lacking *aggR*) were present among cases and controls. The majority of EPEC were atypical EPEC [n = 76 (92%) and only 6 were typical EPEC, 2 among cases and 4 among controls (Table 6). STEC was only detected among cases (0.7%). Other *E*. *coli* pathotypes were detected in lower proportion (Table 5). Logistic regression analysis showed that there was no significant association between *E*. *coli* pathotypes and cases. One exception was EPEC that was associated with controls (Table 4). Out of 27 subjects who had ETEC, 12 had heat stable toxin ETEC (ST-ETEC), and all of them were AGE cases (Table 6). The difference between cases and controls with respect to ST-ETEC infection was statistically significant (*p* = <0.001).

**Table 6:** Distribution of *Escherichia coli* pathotypes and virulence genes among cases and controls.

Pathotype (n)^a^	Subtypes	Genes	Cases		Controls	
			n	%	n	%
ETEC (27)	ST-ETEC	*st*	7	41.2	0	-
	nonST-ETEC	*lt*	5	29.4	10	100
	ST-ETEC	*st*, *lt*	5	29.4	0	-
EAEC (115)^a^	Typical EAEC	*aggR* with or without *aaiC*	38	65.5	38	66.7
	Atypical EAEC	*aaiC*, with negative aggR	20	34.5	19	33.3
EPEC (82)	Atypical EPEC	*eae*^b^	29	93.5	47	92.2
	Typical EPEC	*eae*, *bfpA*^c^	2	6.5	4	7.8
DAEC (6)		*daaE*	4	100	2	100
EIEC (3)		*ipaH*, *ipaD*	1	100	2	100
STEC (3)		*eae*, *stx1 and/or stx2*	3	100	0	-
Emergent *E*. *coli* pathotypes	EAEC/ETEC (1)	*aggR*, *aaiC*, *st*	1	25	0	-
	EAEC/EPEC (1)	*aggR*, *aaiC*, *eae*, *bfpA*	1	25	0	-
	BF-EIEC (2)	*ipaH*, *ipaD*, O96:H19^d^	2	50	0	-

^a^The total number of subjects positive for EAEC were 115, however the total number of typical EAEC and atypical EAEC was 122 because both typical and atypical EAEC were detect in the stool of a total of 5 subjects;

^b^ eae: this genotype corresponds to atypical ETEC;

^c^*eae*, *bfpA* genotype corresponds to typical EPEC;

^d^ BF-EIEC is an emergent EIEC strain with unusual serotype.

Abbreviations: ETEC: Enterotoxigenic *E*. *coli*; ST-ETEC: Heat stable toxin ETEC; EAEC: Enteroaggregative *E*. *coli*; EPEC: Enteropathogenic *E*. *coli*; EIEC: Enteroinvasive E. coli; and STEC: Shiga toxin-producing *E*. *coli*; DAEC: Diffusely adherent *E*. *coli*; BF-EIEC: Biofilm-forming enteroinvasive *E*. *coli*.

In addition to classical *E*. *coli* pathotypes, we identified four cases with stool positive for three novel emergent *E*. *coli* pathotypes (Table 5). Two cases had emergent *E*. *coli* pathotypes with genotypes from two different *E*. *coli* pathotypes (Table 6). One emergent pathogen was designated EAEC-EPEC (*aggR*, *aaiC*, *eae*, and *bfpA*) because it was positive for EAEC specific virulence genes (*aggR* and *aaiC*) and for EPEC specific genes (*bfpA*). The second emergent pathogen was designated EAEC-ETEC (*aggR*, *aaiC*, *st*) because it was also positive for EAEC genes (*aggR*, *aaiC*) and positive for ETEC specific toxin gene (*st*) (Table 6). The remaining two cases were both infected with a third emergent *E*. *coli* pathotype designated as biofilm-forming EIEC, based on the unusual O96:H19 serotype (Table 6).

## Discussion

This is, to our knowledge, the first large, prospective, multicenter, case-control study on AGE conducted in Bucaramanga, Colombia. The study evaluated 24 different enteric microorganisms among children less than 5 years of age with or without AGE. Overall, about 30% of AGE cases could be attribute to a viral pathogen and 6% to a bacterial pathogen. Also, we identified three emergent *E*. *coli* pathotypes not previously described in this geographic region. In contrast with studies conducted in low-income nations [3], no deaths related or unrelated to AGE were reported in this study based on follow-ups at 2 weeks and 2 months after enrollment.

The most prevalent agents associated with AGE in our study were norovirus, rotavirus, adenovirus, and astrovirus. Similar data was reported in the US and in Brazil [31,32]. In our study, norovirus surpassed rotavirus as a leading cause of AGE, in similar fashion as reported in several industrialized and middle-income countries, including US, Brazil and Peru [28,33–36]. This is in contrast with data reported from Africa and South East Asia where rotavirus, *Cryptosporidium*, ETEC, and *Shigella*, were the most common enteric pathogens associated with AGE [3]. Our study adds relevance to the role that enteric viruses, other than rotavirus, play in childhood AGE, including norovirus, adenovirus, astrovirus and sapovirus. In addition, *Campylobacter* spp., *Salmonella* spp., *Cryptosporidium*, and ST-ETEC were also associated with AGE. Except for *Cryptosporidium*, other intestinal parasites were not associated with AGE. Whether specific parasites were not pathogenic, or whether hosts were resistant to parasitic infection, is not clear and further studies will be necessary to elucidate this question.

As a pathogenic group, *E*. *coli* pathotypes were the most common infectious agents among cases (28%) and controls (26%). EAEC were the most common *E*. *coli* pathotype in this study. Based on virulence gene detection, they included typical (*aggR+*) and atypical (*aacA+*, *aggR-*) EAEC, none of which were associated with AGE. Lack of EAEC association with AGE was recognized in studies conducted in Peru, Brazil, and in several countries in Africa and Asia, where EAEC infection was reported as subclinical [37,38]. Other studies indicated that only a fraction of EAEC, carrying unique virulence factors, are associated with diarrhea [39,40]. Most EPEC detected in this study were atypical EPEC, defined as *E*. *coli* strains carrying the LEE pathogenicity island and lacking the major fimbrial subunit (*bfpA*) gene of the bundle-forming pilus. Atypical EPEC strains have a highly diverse genetic makeup and a large variety of serotypes, and their role in human host pathogenesis is at present unclear [41]. ETEC was the only *E*. *coli* pathotype associated with AGE, corroborating prior findings reported by our team in the city of Cartagena, Colombia [14] and other low- and middle-income countries in Latin America, Africa and South East Asia [3,42–44]. More importantly, ST toxin-carrying ETEC were isolated in all cases, indicating that ST-containing ETEC, rather than LT-, are associated with AGE in children. This association of ST-ETEC with severe diarrheal disease was also reported in other low- and middle-income countries [45,46].

Our study identified three emergent *E*. *coli* pathogens, not previously reported in Colombia. Two emergent *E*. *coli* pathotypes had genotypes from two different *E*. *coli* pathotypes, and they were designated EAEC/EPEC and EAEC/ETEC. These organisms were isolated from two children with moderate to severe AGE and they carry the corresponding virulence genotypes (Table 6). Although these novel pathotypes were detected in children with AGE, this study was not designed to understand their role in AGE, the modes of transmission, or the reservoirs. More studies on these emergent pathotypes are necessary to identify the virulence genes repertoire and the role they may play in the pathogenesis of AGE. A third emergent pathogen, designated BF-EIEC, had an unusual O96:H19 serotype, and was identified in two children with AGE. The BF-EIEC strains form strong biofilms on inert surfaces and epithelial cells [47]. EIEC clinical isolates with identical serotype were recently recognized as emergent pathogens associated with severe AGE outbreaks and sporadic cases of diarrhea in Europe [48,49] and most recently in Uruguay [50].

Our study found higher co-infection rates among cases (28%) than controls (14%), this difference was statistically significant (*p*-value <0.001). Co-infection was also reported in low- and middle-income countries from all continents [3,51,52], yet it was less common among children in the US [53]. AGE etiology determination is difficult when cases and controls have co-infections. Multiple variables may contribute to disease status in the presence of co-infections including: i) pathogens combinations may be more pathogenic than individual pathogens; ii) only one of the organisms may be pathogenic; iii) pathogen load may contribute to AGE. Our data suggest that the presence of more than one pathogen per subject was associated with AGE. The detection of two or more enteropathogens associated with AGE was also reported in a case control study in Mexican children [51]. In addition to co-infections, there was a high infection rate among cases (71%) and controls (54%) in all age groups. This data suggests that this population is acquiring enteric pathogens at early age and remain colonized for a long time. The main sources of infections or co-infections among children in this study are unknown, as this study did not evaluate water or food product contamination as infection sources. We believe that food products may be important sources of enteric pathogens and vehicles of infection transmission as previously reported in Colombia [17,54].

Epidemiological factors that may contribute to differences in pediatric AGE enteric pathogen detection between our study site in Colombia and low-income countries, may include access to health care services, high rotavirus vaccine coverage (90%), access to clean water (64%) and mothers with primary school education level or higher (>80%), compared to low income country data [3]. The lower rate of *Shigella* spp., *Campylobacter* spp., and *G*. *intestinalis* infections in this study maybe explained, in part, by satisfactory hygiene conditions in the community, and to a lesser extent, by the use of antibiotics prior to enrollment among 12% of cases. This study did not report AGE-associated deaths, nor deaths in controls after follow up phone calls at 14 days or 60 days post-enrollment. Our data is consistent with the low AGE-associated mortality in children less than 5 years of age in Colombia (3.0 per 100,000), which is similar to the mortality reported in other middle-income Latin American countries, including Brazil, and Argentina as well as high-income countries such the USA or Saudi Arabia, where AGE-associated mortality ranges from 1.0 to 4.0 per 100,000 children [55–57].

This study has some limitations. Only diagnostic tools available to laboratories in low- and middle-income countries, including stool bacterial cultures, ELISA, and PCR, were used. Metagenomics or whole-genome sequencing technology, not available for surveillance purposes in this region, was not used. Stool culture might have lower sensitivity for bacterial pathogen detection compared to culture independent diagnostics tests as shown in African and South East Asian studies where detection of *Shigella*, *EIEC*, *ETEC-ST* and *Campylobacter* increased with use of quantitative PCR compared to stool culture [58]. However, stool cultures were essential in our study to detect the presence of novel *E*. *coli* pathogens. Bacterial culture testing was also critical to identify individual *E*. *coli* pathotypes or subtypes within the same subject. These findings would have been impossible using conventional PCR techniques. The cases were recruited from outpatient, inpatient and emergency departments while controls were recruited from outpatient settings only. This means that controls might be healthier at baseline compared to cases. Even though we looked at the disaggregated data based on age groups, our study was not powered to detect a difference based on age groups. The relatively low proportion of hospitalized cases (12%) in this study of moderate to severe AGE may not represent well the severe side of AGE. Further studies on severe AGE may reveal additional epidemiological and microbiological factors of this disease. This study was conducted in an urban area where basic needs are satisfied for most of the population, accordingly, data from this study is not representative of rural areas in Colombia or other LMIC in the region where unsatisfied needs are common, including deficiencies in the potable water, sanitation, and hygiene [59].

Rotavirus was the second most common viral agent detected among cases of moderate to severe AGE. This data may be in contrast with recent reports indicating that rotavirus immunization is effective in preventing severe AGE in Latin America and the Caribbean [60,61]. A possible explanation to this discrepancy is that the number of rotavirus infections in our study occurred in ambulatory settings where vaccine is less likely to have a high impact. Also, infections in our study may include emerging rotavirus genotypes that may be less likely to be prevented by current RV1 or RV5 rotavirus vaccines [62]. Alternatively, the high number of rotavirus infection among cases may be the result of rotavirus sheading not associated directly with AGE, since rotavirus vaccines do not prevent rotavirus infection or secretion as previously described [63]. We believe that more studies will be necessary to determine whether the high number of rotavirus infections in this population is of major concern or, on the contrary, the number of rotavirus infections may include asymptomatic rotavirus infections not associated with AGE. The number of norovirus G1 infections detected in cases and controls in this study was identical. Since norovirus are shed for 3 to 4 weeks after onset of AGE [64] and control subjects were enrolled as healthy children with no history of AGE for the last 10 days, it is possible that some of the control subjects that tested positive for norovirus were a result of recent cases of norovirus AGE. This limitation of the study may explain, at least in part, the lack of association of norovirus GI with AGE.

In summary, this study from Bucaramanga, Colombia, South America, showed that norovirus, rotavirus, adenovirus and sapovirus were the most common infectious agents associated with moderate to severe AGE in children < 5 years of age. *E*. *coli* pathotypes were the most common pathogens detected in both cases and controls, yet ST-containing ETEC was the only one associated with AGE. The higher proportion of pathogens among cases, as either single infections or co-infections, was associated to AGE. Furthermore, this study reports for the first time the detection of three unique emergent *E*. *coli* pathogens among cases in Colombia, designated EAEC/ETEC, EAEC/EPEC and the BF-EIEC.

## Supporting information

S1 Fig Flowchart of microbioal detection protocols. Stools from cases and controls were processed for detection of bacterial, viral, and parasitic microorganisms. Molecular-based techniques, immunological assays, microbiological assays, and microscopy protocols were used.
(TIF)

S1 Table Primer and probes for the conventional multiplex PCR and real-time RT-PCR. (DOCX)

S2 Table Reaction Mix for norovirus, astrovirus, sapovirus and Campylobacter PCR (DOCX)

S3 Table Proportion of single infection and co-infection among cases of AGE and controls. (DOCX)

S4 Table Prevalence of single pathogens or Co-infection and association with moderate to severe diarrhea (DOCX)

S5 Table STROBE checklist. (DOCX)

S6 Table *E*. *coli* clinical isolates from cases and controls. (XLSX)
