# Supplementary figures and images for: Etiology of acute gastroenteritis among children less than 5 years of age in Bucaramanga, Colombia: A case-control study
Source: PLoS Negl Trop Dis. 2020 Jun 30;14(6):e0008375. doi: 10.1371/journal.pntd.0008375 (PMC7357789; doi:10.1371/journal.pntd.0008375)

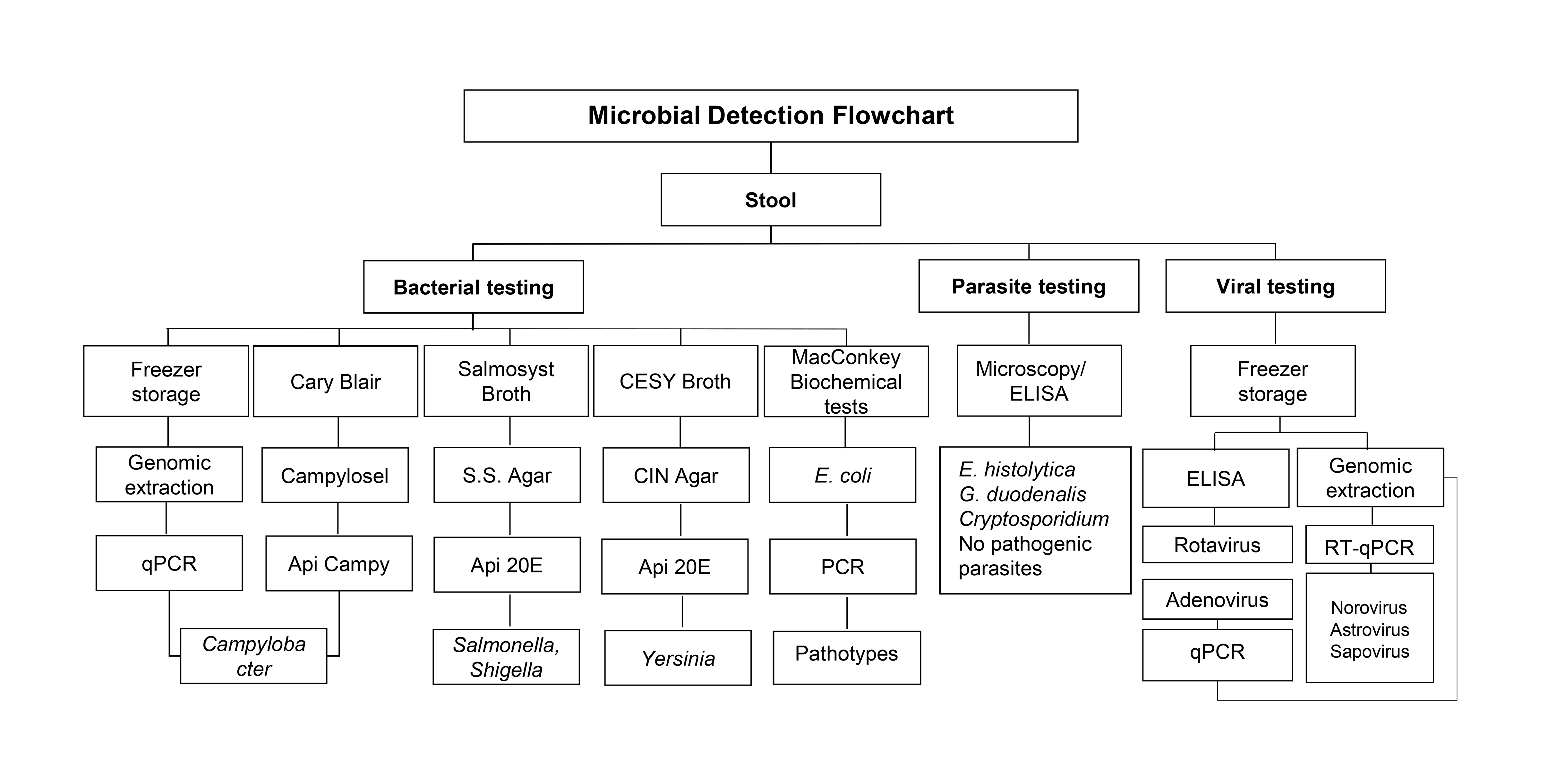

Supplement: S1 Fig — Stools from cases and controls were processed for detection of bacterial, viral, and parasitic microorganisms. Molecular-based techniques, immunological assays, microbiological assays, and microscopy protocols were used. (TIF) [file pntd.0008375.s001.tif]
