# Supplementary material for: Etiology of acute gastroenteritis among children less than 5 years of age in Bucaramanga, Colombia: A case-control study
Source: PLoS Negl Trop Dis. 2020 Jun 30;14(6):e0008375. doi: 10.1371/journal.pntd.0008375 (PMC7357789; doi:10.1371/journal.pntd.0008375)
Supplement: S1 Table — (DOCX) [file pntd.0008375.s002.docx]

STable 1. Primer and probes for the conventional multiplex PCR and real-time RT-PCR

| **Organisms** | **Primers** | **Target** | **Primer Sequences (5’-3’)** | | **References** |  |
| --- | --- | --- | --- | --- | --- | --- |
| DAEC | daaE-F | *daaE* | GAA CGT TGG TTA ATG TGG GGT AA | | 65 | [1] |
|  | daaE-R |  | TAT TCA CCG GTC GGT TAT CAG T | |  |  |
| EAEC | aaiC-F | *aaiC* | ATT GTC CTC AGG CAT TTC AC | | 66 | [2] |
|  | aaiC-R |  | ACG ACA CCC CTG ATA AAC AA | |  |  |
|  | aggR-F | *aggR* | GTA TAC ACA AAA GAA GGA AGC | | 67 | [3] |
|  | aggR-R |  | ACA GAA TCG TCA GCA TCA GC | |  |  |
| EIEC | ipaH-F | *ipaH* | CTC GGC ACG TTT TAA TAG TCT GG | | 65 | [2] |
|  | ipaH-R |  | GTG GAG AGC TGA AGT TTC TCT GC | |  |  |
| EPEC | Bfp-F | *bfpA* | AAT GGT GCT TGC GCT TGC TGC | | 67 | [3] |
|  | Bfp-R |  | GCC GCT TTA TCC AAC CTG GTA | |  |  |
|  | eae384-F | *eae* | GAC CCG GCA CAA GCA TAA GC | | 66 | [2] |
|  | eae384-R |  | CCA CCT GCA GCA ACA AGA GG | |  |  |
|  | eae917-F | *eae* | CTG AAC GGC GAT TAC GCG AA | | 67 | [3] |
|  | eae917-R |  | CGA GAC GAT ACG ATC CAG | |  |  |
| ETEC | LT-F | *Elt* | CAC ACG GAG CTC CTC AGT C | | 66 | [2] |
|  | LT-R |  | CCC CCA GCC TAG CTT AGT TT | |  |  |
|  | ST-F | *est* | GCT AAA CCA GTA G/AGG TCT TCA AAA | | 66 | [2] |
|  | ST-R |  | CCC GGT ACA G/AGC AGG ATT ACA ACA | |  |  |
| STEC | eae917-F | *eae* | CTG AAC GGC GAT TAC GCG AA | | 67 | [3] |
|  | eae917-R |  | CGA GAC GAT ACG ATC CAG | |  |  |
|  | eae384-F | *eae* | GAC CCG GCA CAA GCA TAA GC | | 66 | [2] |
|  | eae384-R |  | CCA CCT GCA GCA ACA AGA GG | |  |  |
|  | stx1-F | *stx1* | ATA AAT CGC CAT TCG TTG ACT AC | | 66 | [2] |
|  | stx1-R |  | AGA ACG CCC ACT GAG ATC ATC | |  |  |
|  | stx2-F | *stx2* | GGC ACT GTC TGA AAC TGC TCC | | 66 | [2] |
|  | stx2-R |  | TCG CCA GTT ATC TGA CAT TCT G | |  |  |
| *E. coli* | uidA-F | *uidA* | GCGTCTGTTGACTGGCAGGTGGTGG | | 12 | [4] |
|  | uidA-R |  | GTTGCCCGCTTCGAAACCAATGCCT | |  |  |
| *Campylobacter* | cadF-F | *cadF* | CTGCTAAACCATAGAAATAAAATTTCTCAC | | 68 | [5] |
|  | cadF-R |  | CTTTGAAGGTAATTTAGATATGGATAATCG | |  |  |
| Norovirus GI | Cog 1F | GI | CGYTGGATGCGNTTYCATGA | | 69 | [6] |
|  | Cog 1R |  | CTTAGACGCCATCATCATTYAC | |  |  |
|  | Cog 2R |  | TCGACGCCATCTTCATTCACA | | 69 | [6] |
| MS phage | MS2.F | 289–387 | TGGCACTACCCCTCTCCGTATTCACG | | 27 | [7] |
|  | MS2.R |  | GTACGGGCGACCCCACGATGAC | |  |  |
| Astrovirus | AsFF | ORF1b | GGC CAG ACT CAC AGA AGA GCA | | 13 | [8] |
|  | AsFr |  | GTC CTG TGA CAC CTT GTT TCC TGA | |  |  |
| Sapovirus | SaV124F | ORF1 | GAY CAS GCT CTC GCY ACC TAC | | 70 | [9] |
|  | SaV1F |  | TTG GCC CTC GCC ACC TAC | |  |  |
|  | SaV5F |  | TTT GAA CAA GCT GTG GCA TGC TAC | | 70 | [9] |
|  | SaV1245R |  | CCC TCC ATY TCA AAC ACT A | |  |  |
| Adenovirus | JTVFF | Hexon | AAC TTT CTC TCT TAA TAG ACG CC | | 71 | [10] |
|  | JTVFR |  | AGG GGG CTA GAA AAC AAA A | |  |  |
| **Organisms** | Probes | Target | Probe Sequences (5’-3’) | | Reference |  |
| *Campylobacter* | cadFP | cadF | [HEX]-CATTTTGACGATTTTTGGCTTGA-[BHQ2] | 68 | | [5] |
| Norovirus GI | Ring 1E | GI | [6-FAM]-AGATYGCGRTCYCCTGTCCA-[Tamra] | 69 | | [6] |
| Norovirus GII | Ring 2 | GII | [CY5]-TGGGAGGGCGATCGCAATCT-[BHQ2] | 69 | | [6] |
| MS phage | MS2.P probe | neucleotide 289–387 | [5-HEX]-CACATCGATAGATCAAGGTGCCTACAAGC-[BHQ1] | 27 | | [7] |
| Astrovirus | AstZFb | ORF1b | [5-HEX]-CCA TCG CAT TTG GAG GGG AGG ACC AGC GA-[BHQ1] | 13 | | [8] |
| Sapovirus | SaV124TP | ORF1 | [6-FAM]-CCR CCT ATR AAC CA-[MGB-NQF] | 70 | | [9] |
|  | SaV5TP | ORF1 | [6-FAM]-TGC CAC CAA TGT ACC A-[MGB-NQF] | 70 | | [9] |
| Adenovirus | JTVFAP | Hexon | [6-FAM]-CGA AGA GTG CCC GTG TCA GC-[BHQ1] | 71 | | [10] |

Abreviations. DAEC = Diffusely adherent *E. coli*; EAEC = Enteroaggregative *E. coli*; EIEC = Enteroinvasive *E. coli*; EPEC = Enteropathogenic *E. coli*; ETEC = Enterotoxigenic *E. coli;* and STEC = Shiga toxin-producing *E. coli*; GI: Norovirus GI ORF1–ORF2 junction DNA; GII: Norovirus GII ORF1–ORF2 junction DNA.

References for S1 Table

1. Vidal M, Kruger E, Durán C, Lagos R, Levine M, Prado V, et al. Single multiplex PCR assay to identify simultaneously the six categories of diarrheagenic *Escherichia coli* associated with enteric infections. J Clin Microbiol. 2005;43: 5362–5365. doi:10.1128/JCM.43.10.5362-5365.2005

2. Panchalingam S, Antonio M, Hossain A, Mandomando I, Ochieng B, Oundo J, et al. Diagnostic microbiologic methods in the GEMS-1 case/control study. Clin Infect Dis. 2012;55 Suppl 4: S294-302. doi:10.1093/cid/cis754

3. Aranda KRS, Fabbricotti SH, Fagundes-Neto U, Scaletsky ICA. Single multiplex assay to identify simultaneously enteropathogenic, enteroaggregative, enterotoxigenic, enteroinvasive and Shiga toxin-producing *Escherichia coli* strains in Brazilian children. FEMS Microbiol Lett. 2007;267: 145–150.

4. Gomez-Duarte OG, Romero-Herazo YC, Paez-Canro CZ, Eslava-Schmalbach JH, Arzuza O. Enterotoxigenic *Escherichia coli* associated with childhood diarrhoea in Colombia, South America. J Infect Dev Ctries. 2013;7: 372–381.

5. Platts-Mills JA, Liu J, Gratz J, Mduma E, Amour C, Swai N, et al. Detection of *Campylobacter* in stool and determination of significance by culture, enzyme immunoassay, and PCR in developing countries. J Clin Microbiol. 2014;52: 1074–1080. doi:10.1128/JCM.02935-13

6. Trujillo AA, McCaustland KA, Zheng D-P, Hadley LA, Vaughn G, Adams SM, et al. Use of TaqMan real-time reverse transcription-PCR for rapid detection, quantification, and typing of norovirus. J Clin Microbiol. 2006;44: 1405–1412. doi:10.1128/JCM.44.4.1405-1412.2006

7. Rolfe KJ, Parmar S, Mururi D, Wreghitt TG, Jalal H, Zhang H, et al. An internally controlled, one-step, real-time RT-PCR assay for norovirus detection and genogrouping. J Clin Virol. 2007;39: 318–321. doi:10.1016/j.jcv.2007.05.005

8. Grant L, Vinjé J, Parashar U, Watt J, Reid R, Weatherholtz R, et al. Epidemiologic and clinical features of other enteric viruses associated with acute gastroenteritis in American Indian infants. J Pediatr. 2012;161: 110-115.e1. doi:10.1016/j.jpeds.2011.12.046

9. Oka T, Katayama K, Hansman GS, Kageyama T, Ogawa S, Wu F-T, et al. Detection of human sapovirus by real-time reverse transcription-polymerase chain reaction. J Med Virol. 2006;78: 1347–1353. doi:10.1002/jmv.20699

10. Lyman WH, Walsh JF, Kotch JB, Weber DJ, Gunn E, Vinjé J. Prospective study of etiologic agents of acute gastroenteritis outbreaks in child care centers. J Pediatr. 2009;154: 253–257. doi:10.1016/j.jpeds.2008.07.057
