# Supplementary material for: Etiology of acute gastroenteritis among children less than 5 years of age in Bucaramanga, Colombia: A case-control study
Source: PLoS Negl Trop Dis. 2020 Jun 30;14(6):e0008375. doi: 10.1371/journal.pntd.0008375 (PMC7357789; doi:10.1371/journal.pntd.0008375)
Supplement: S2 Table — (DOCX) [file pntd.0008375.s003.docx]

STable 2. Reaction Mix for norovirus, astrovirus, sapovirus and *Campylobacter* PCR

| **Reaction Mix for GI/GII Norovirus Multiplex RT-qPCR Assay** | | |
| --- | --- | --- |
| **Component** | Volume per reaction (μl) | Final concentration |
| 2X RT-PCR buffer* | 12.5 | 1X |
| Nuclease-free water* | 1.08 | n/a |
| Detection Enhancer* | 1.67 | n/a |
| Cog1F (10 μM) | 1 | 400 nM |
| Cog1R (10 μM) | 1 | 400 nM |
| Ring 1E (10 μM) | 0.5 | 200 nM |
| Cog2F (10 μM) | 1 | 400 nM |
| Cog2R (10 μM) | 1 | 400 nM |
| Ring 2 (10 μM) | 0.5 | 200 nM |
| MS2.F (10 μM) | 0.25 | 100 nM |
| MS2.R (10 μM) | 0.25 | 100 nM |
| MS2.P (10 μM) | 0.25 | 100 nM |
| 25X RT-PCR enzyme* | 1 | 1x |
| RNA sample | 3 |  |
|  |  |  |
| **Reaction Mix Astrovirus/Sapovirus Duplex RT-qPCR Assay** | | |
| **Component** | Volume per reaction (μl) | Final concentration |
| 2X RT-PCR buffer* | 12.5 | 1X |
| Nuclease-free water* | 2.5 | n/a |
| AsFF (10 μM) | 0.6 | 250 nM |
| AsFR (10 μM) | 0.6 | 250 nM |
| AstZFB (10 μM) | 0.25 | 100 nM |
| SaV 124F (10 μM) | 1 | 400 nM |
| SaV 1F (10 μM) | 1 | 400 nM |
| SaV 5F (10 μM) | 1 | 400 nM |
| SaV 1245R (10 μM) | 1 | 400 nM |
| SaV124TP (10 μM) | 0.25 | 100 nM |
| SaV 5TP (10 μM) | 0.25 | 100 nM |
| 25X RT-PCR enzyme* | 1 | 1x |
| RNA sample | 3 |  |
|  |  |  |
| Reaction Mix for adenovirus RT-qPCR Assay | | |
| Component | Volume per reaction (μl) | Final concentration |
| 2X RT-PCR buffer* | 12.5 | 1X |
| Nuclease-free water* | 4.33 | n/a |
| Detection Enhancer* | 1.67 | n/a |
| Cog1F (10 μM) | 1 | 400 nM |
| Cog1R (10 μM) | 1 | 400 nM |
| Ring 1E (10 μM) | 0.5 | 200 nM |
| 25X RT-PCR enzyme* | 1 | 1x |
| RNA sample | 3 |  |
|  |  |  |
| **Reaction Mix for *Campylobacter* qPCR Assay** | | |
| **Component** | Volume per reaction (μl) | Final concentration |
| TaqMan Environmental Master Mix 2.0 | 12.5 | 1X |
| Nuclease-free water | 9 | n/a |
| cadF-F (10 μM) | 1 | 400 nM |
| cadF-R (10 μM) | 1 | 400 nM |
| cadF-P (10 μM) | 0.5 | 200 nM |
| DNA sample | 1 |  |
|  |  |  |
| * Included in the Ag-Path Kit | | |
