# Supplementary material for: Etiology of acute gastroenteritis among children less than 5 years of age in Bucaramanga, Colombia: A case-control study
Source: PLoS Negl Trop Dis. 2020 Jun 30;14(6):e0008375. doi: 10.1371/journal.pntd.0008375 (PMC7357789; doi:10.1371/journal.pntd.0008375)
Supplement: S3 Table — (DOCX) [file pntd.0008375.s004.docx]

STable 3. Proportion of single infection and co-infection among cases of AGE and controls.

| **Infection** | **Cases** | |  |  | **Controls** | | **Total** | | ***p*-value** |
| --- | --- | --- | --- | --- | --- | --- | --- | --- | --- |
|  | n | % |  |  | n | % | n | % |  |
| Single | 184 | 42.7 |  |  | 172 | 40.0 | 356 | 41.3 | <0.422 |
| Co-infection | 123 | 28.5 |  |  | 60 | 14.2 | 183 | 21.3 | <0.001 |
| No infection | 124 | 28.8 |  |  | 198 | 46.0 | 322 | 37.4 | <0.001 |
| Total | 431 |  |  |  | 430 |  | 861 |  |  |

*The numbers present on the row designated “Any infection” were not used to calculate total number on any of the columns.
