# Supplementary material for: Etiology of acute gastroenteritis among children less than 5 years of age in Bucaramanga, Colombia: A case-control study
Source: PLoS Negl Trop Dis. 2020 Jun 30;14(6):e0008375. doi: 10.1371/journal.pntd.0008375 (PMC7357789; doi:10.1371/journal.pntd.0008375)
Supplement: S4 Table — (DOCX) [file pntd.0008375.s005.docx]

STable 4. Prevalence of single pathogens or Co-infection and association with moderate to severe AGE

| Pathogen | Status of Co-infection | Case n=431 | Control  n=430 | Crude OR (95% CI) | Adjusted OR  (95 % CI) |
| --- | --- | --- | --- | --- | --- |
| EAEC | Single | 24(5.3%) | 36(8.6%) | 0.62(0.36-1.07) | 0.64(0.37-1.12) |
|  | Co-infection | 34(7.7%) | 21(3.7%) | 2.1(1.11-3.8) | 2.0 (1.01-3.7)* |
| Norovirus | Single | 34(7.9%) | 20(4.7%) | 1.9(1.11-3.5) | 2.1(1-3.9) |
|  | Co-infection | 55(12.8%) | 12(2.8%) | 5.3(2.8-10) | 5.0(2.6-9.6) |
| EPEC | Single | 12(2.8%) | 30(7%) | 0.44(0.21-0.88) | 0.48(0.24-0.99) |
|  | Co-infection | 15(3.5%) | 17(4%) | 0.84(0.41-1.7) | 0.81(0.39-1.6) |
| *E. histolytica* | Single | 14(3.3%) | 20(4.7%) | 0.67(0.33-1.3) | 0.67(0.32-1.36) |
|  | Co-infection | 17(3.9%) | 23(5.3%) | 0.71(0.37-1.3) | 0.65(0.33-1.3) |
| *Blastocystis* | Single | 17(3.9%) | 16(3.7%) | 1.0(0.54-2.1) | 0.98(0.47-2.0) |
|  | Co-infection | 26(6%) | 17(4%) | 1.5(0.83-2.9) | 1.4(0.74-2.0) |
| Sapovirus | Single | 10(2.3%) | 13(3%) | 0.78(0.33-1.8) | 0.75(0.32-1.7) |
|  | Co-infection | 16(3.7%) | 5(1.2%) | 3.2(1.18-8.9) | 2.4(0.85-6.9) |
| Rotavirus | Single | 18(4.2%) | 1(0.2%) | 19.7(2.6-148) | 19.3(2.5-147) |
|  | Co-infection | 23(5.3%) | 1(0.2%) | 25.2(3.3-187) | 25.6(3.3-191) |
| *Giardia duodenalis* | Single | 6 (1.4%) | 11 (2.6%) | 0.54(0.19-1.4) | 0.55(0.2-1.5) |
|  | Co-infection | 18(4.2%) | 14(3.3%) | 1.2(0.62-2.6) | 1.2(0.58-2.6) |
| *Cryptosporidium* | Single | 4(0.9%) | 2(0.4%) | 2.0 (0.37-11) | 2.2(0.4-12) |
|  | Co-infection | 8(1.8%) | 1(0.2%) | 8.0 (1.0-65) | 8.3(1.0-67)* |
| Astrovirus | Single | 8(1.8%) | 3(0.7 %) | 2.7(0.72-10.3) | 2.7(0.71-10.4) |
|  | Co-infection | 11(2.6%) | 4(0.9%) | 2.8(0.89-8.9) | 2.6(0.82-8.5)* |
| *E. coli* Pathotypes | Single | 46(10.7%) | 75(17.4%) | 0.61(0.41-0.91) | 0.64(0.43-0.97) |
|  | Co-infection | 67(15.6%) | 39(9%) | 1.7(1.12-2.6) | 1.7(1.1-2.6) |
| *Campylobacter* | Single | 9(2.1%) | 5(1.2%) | - | - |
|  | Co-infection | 6(1.4%) | 0 | - | - |
| *Salmonella* | Single | 5(1.2%) | 2(0,5%) | - | - |
|  | Co-infection | 7(1.6%) | 0 | - | - |
| *Adenovirus* | Single | 7(1.6%) | 0 | - | - |
|  | Co-infection | 7(1.6%) | 1(0.2) | - | - |

Abbreviations: EAEC: enteroaggregative *E. coli*; EPEC: enteropathogenic *E. coli*.

Pathogens not listed were present only as co-infection.

* OR and CI values above 1.0 that indicate association with AGE.
