# Supplementary material for: Etiology of acute gastroenteritis among children less than 5 years of age in Bucaramanga, Colombia: A case-control study
Source: PLoS Negl Trop Dis. 2020 Jun 30;14(6):e0008375. doi: 10.1371/journal.pntd.0008375 (PMC7357789; doi:10.1371/journal.pntd.0008375)
Supplement: S5 Table — (DOCX) [file pntd.0008375.s006.docx]

**S Table 5.** STROBE checklist.

STROBE Statement—Checklist of items that should be included in reports of ***case-control studies***

|  | Item No | Recommendation |
| --- | --- | --- |
| Title and abstract | 1 | (*a*) Indicate the study’s design with a commonly used term in the title or the abstract. Response: Study’s design indicated in the title. |
|  |  | (*b*) Provide in the abstract an informative and balanced summary of what was done and what was found.  Response: Information on methods and findings is summarized in the abstract. Page2, lines42 to 62. |
| Introduction | | |
| Background/rationale | 2 | Explain the scientific background and rationale for the investigation being reported.  Response: Background and rationale for this study is explained in the introduction section. Page 5, lines 85 to 109. |
| Objectives | 3 | State specific objectives, including any prespecified hypotheses.  Response: The main objectives of this study were to define the etiology of moderate to severe AGE in children less than 5 years old in Bucaramanga, Colombia, a representative mid-size tropical city among middle-income tropical countries in Latin America and to identify novel and emergent *E. coli* pathotypes. See page 6, lines109 to 112. |
| Methods | | |
| Study design | 4 | Present key elements of study design early in the paper.  Response: Study design is described first in the Materials and Methods’ section. Page 7, lines 116 to 136. |
| Setting | 5 | Describe the setting, locations, and relevant dates, including periods of recruitment, exposure, follow-up, and data collection.  Response: Description of setting, locations and relevant dates are described in page 7, lines 116 to 136. Also, there is a description of sites in supplementary materials section page 1, first paragraph. |
| Participants | 6 | (*a*) Give the eligibility criteria, and the sources and methods of case ascertainment and control selection. Give the rationale for the choice of cases and controls.  Response: The eligibility criteria for cases and controls is provided in supplementary table 1, located in supplementary materials page 3. The rational for selection of cases was to study the epidemiology and the etiology of acute gastroenteritis (AGE) in children with AGE and compare it with children with no AGE. |
|  |  | (*b*) For matched studies, give matching criteria and the number of controls per case  Response: Matching criteria: Matched to cases for age. Age matching is ±2 months for 0-11 months, ±4 months for 12-59 months. (can not exceed the stratum boundaries of the case). This information is described in Supplementary Table 1. |
| Variables | 7 | Clearly define all outcomes, exposures, predictors, potential confounders, and effect modifiers. Give diagnostic criteria, if applicable  Response: Variables for this study included epidemiological factors as described in Table 1. AGE variables including clinical manifestations such as diarrhea, vomiting, fever, abdominal pain, dehydration, blood in the stools. This is described in the supplementary data, page 1, second paragraph.  Enteric pathogen detection, infection, co-infection variables were described in page 13, lines 243 to 258.  Type of enteric pathogen detection was presented in pages 14-15, lines 272 to289.  Detection of diarrheagenic E. coli is described in page 15, lines 290 to 304.  Detection of emergent diarrheagenic *E. coli* pathotypes is described in page 16, lines 305 to 311. |
| Data sources/ measurement | 8* | For each variable of interest, give sources of data and details of methods of assessment (measurement). Describe comparability of assessment methods if there is more than one group  Response: Epidemiological variables (age, sex, socioeconomic status, education, health insurance, water sources, etc) were obtained from questionnaire. This is described in the material and methods section, page 8, lines 138 to 145. More information is presented in supplementary data, page 1, second paragraph. |
| Bias | 9 | Describe any efforts to address potential sources of bias  Response: This is address in the statistical methods section, page 10, lines 188 to 194. |
| Study size | 10 | Explain how the study size was arrived at  Response: Sample size calculations were initially based on a balanced design with 300 cases and 300 controls, which was subsequently enriched to 430 in each group to observe more emergent E. coli pathogens. The power to detect significant associations is impacted by the underlying prevalence of a pathogen. With 430 subjects in a group, we had 80% power to detect an odds ratio of 4.8 if the control prevalence was 1% and 80% power to detect an odds ratio of 1.7 if the control prevalence was 20%. All calculations used a 0.05 level of significance. All statistical tests were two-sided. This is described in page 9-10, lines 177 to 183 |
| Quantitative variables | 11 | Explain how quantitative variables were handled in the analyses. If applicable, describe which groupings were chosen and why  Response: Quantitative variables were analysed as described in Statistical methods, page 10, lines 184 to 189. |
| Statistical methods | 12 | (*a*) Describe all statistical methods, including those used to control for confounding |
|  |  | (*b*) Describe any methods used to examine subgroups and interactions |
|  |  | (*c*) Explain how missing data were addressed |
|  |  | (*d*) If applicable, explain how matching of cases and controls was addressed |
|  |  | (*e*) Describe any sensitivity analyses  Response: A section on statistical methods is presented in Material and Methods pages 9-11, lines 177 to 212. |
| Results | | |
| Participants | 13* | (a) Report numbers of individuals at each stage of study—eg numbers potentially eligible, examined for eligibility, confirmed eligible, included in the study, completing follow-up, and analysed  Response: During the 18 months study period, 1,511 children were screened and 861 (57.0%) agreed to participate, including 431 cases of AGE and 430 controls (Figure 1). The baseline characteristics were similar in terms of age, gender, race, income, caregiver education and nutritional status (Table 1). In contrast, handwashing with water and soap was more common in controls (99%) and exposure to sick contacts with diarrhea was more common in cases (17 %) (Table 1). We recruited 12 % (52/431) of cases from inpatient settings and the remaining were recruited from either outpatient clinics or emergency departments. This information is reported in page 12 lines 221 to 228. |
|  |  | (b) Give reasons for non-participation at each stage.  Response: Reasons for non-participation at each stage is provided in figure 2. |
|  |  | (c) Consider use of a flow diagram.  Response: Figure 2 with information about participation is shown in a flow diagram. |
| Descriptive data | 14* | (a) Give characteristics of study participants (eg demographic, clinical, social) and information on exposures and potential confounders  Response: Table 1 provides characteristics of study participants. |
|  |  | (b) Indicate number of participants with missing data for each variable of interest.  Data was recorded for all participants on variables of interest. |
| Outcome data | 15* | Report numbers in each exposure category, or summary measures of exposure  Response: This information is in Clinical features of AGE cases, page 12, lines 230 to 241. Additional information is presented in section called infections and co-infections among cases and controls, page 13, lines 243 to 270. |
| Main results | 16 | (*a*) Give unadjusted estimates and, if applicable, confounder-adjusted estimates and their precision (eg, 95% confidence interval). Make clear which confounders were adjusted for and why they were included.  Response: See results section under demographics of study population in page 12, lines 222 to 228. Also, in table 1. |
|  |  | (*b*) Report category boundaries when continuous variables were categorized.  Response: This is described in in page 10, lines 184 to 186. |
|  |  | (*c*) If relevant, consider translating estimates of relative risk into absolute risk for a meaningful time period.  Response: Not applicable. |

| Other analyses | 17 | Report other analyses done—eg analyses of subgroups and interactions, and sensitivity analyses  Response: We calculated the attributable fraction (risk) for each pathogen. See section in page 11 lines 205 to 212. |
| --- | --- | --- |
| Discussion | | |
| Key results | 18 | Summarise key results with reference to study objectives  Response: This information is reported in page 17, lines 314 to 324. |
| Limitations | 19 | Discuss limitations of the study, taking into account sources of potential bias or imprecision. Discuss both direction and magnitude of any potential bias  Response: This information is presented in page 20-21, lines 398 to 417. |
| Interpretation | 20 | Give a cautious overall interpretation of results considering objectives, limitations, multiplicity of analyses, results from similar studies, and other relevant evidence  Response: Data presented in page 21, lines 418 to 426. |
| Generalisability | 21 | Discuss the generalisability (external validity) of the study results  Response: Data on this is presented in page 21, lines 412 to 417. |
| Other information | | |
| Funding | 22 | Give the source of funding and the role of the funders for the present study and, if applicable, for the original study on which the present article is based  Response: This section is in page 22, lines 435 to 440. |

*Give information separately for cases and controls.

**Note:** An Explanation and Elaboration article discusses each checklist item and gives methodological background and published examples of transparent reporting. The STROBE checklist is best used in conjunction with this article (freely available on the Web sites of PLoS Medicine at http://www.plosmedicine.org/, Annals of Internal Medicine at http://www.annals.org/, and Epidemiology at http://www.epidem.com/). Information on the STROBE Initiative is available at http://www.strobe-statement.org.
